# P-1611. Longitudinal Measurement of Peripheral Blood Cytokines and COVID-19 Severity and Long COVID Outcomes in the EPIC3 Study

**DOI:** 10.1093/ofid/ofaf695.1789

**Published:** 2026-01-11

**Authors:** Calen Mendall, Xumin Li, Vivek Pakanati, Cindy H Liu, Tracy Wang, Daniel Morelli, Anna Korpak, Aaron Baraff, Stuart N Isaacs, Amy Vittor, Kyong-Mi Chang, Elizabeth Le, Nicholas L Smith, Jennifer S Lee, Jennifer M Ross, Javeed Shah

**Affiliations:** VA Puget Sound Health Care System, Department of Epidemiology at University of Washington, Seattle, Washington; VA Puget Sound Health Care System, Department of Epidemiology at University of Washington, Seattle, Washington; VA Puget Sound Health Care System, Seattle, Washington; VA Puget Sound Healthcare System, Seattle, Washington; VA Puget Sound Health Care System, Seattle, Washington; VA Puget Sound Health Care System, Seattle, Washington; VA Puget Sound Health Care System, Seattle, WA, USA, Seattle, Washington; VA Puget Sound Health Care System, Seattle, Washington; Philadelphia VA Medical Center, Philadelphia, Pennsylvania; North Florida/South Georgia Veterans Health System, Division of Infectious Disease and Global Medicine at University of Florida, Gainesville, Florida; Corporal Michael J. Crescenz VA Medical Center, Philadelphia, Pennsylvania; VA Palo Alto Health Care System, Palo Alto, California; VA Puget Sound Health Care System, Department of Epidemiology at University of Washington, Seattle, Washington; VA Palo Alto Health Care System, Division of Endocrinology, Gerontology, and Metabolism at Stanford University, Palo Alto, California; VA Puget Sound Health Care System, Division of Allergy and Infectious Diseases at University of Washington, Seattle, Washington; University of Washington, Seattle, Washington

## Abstract

**Background:**

Immune profile shifts during acute and late SARS-CoV-2 infection may provide insight into the risk of severe COVID-19 and Long COVID. U.S. Veterans are at high risk of severe or persistent COVID-19; associations between these outcomes and immune profiles during acute and late SARS-CoV-2 infection are not currently described in this population.

**Table 1.**
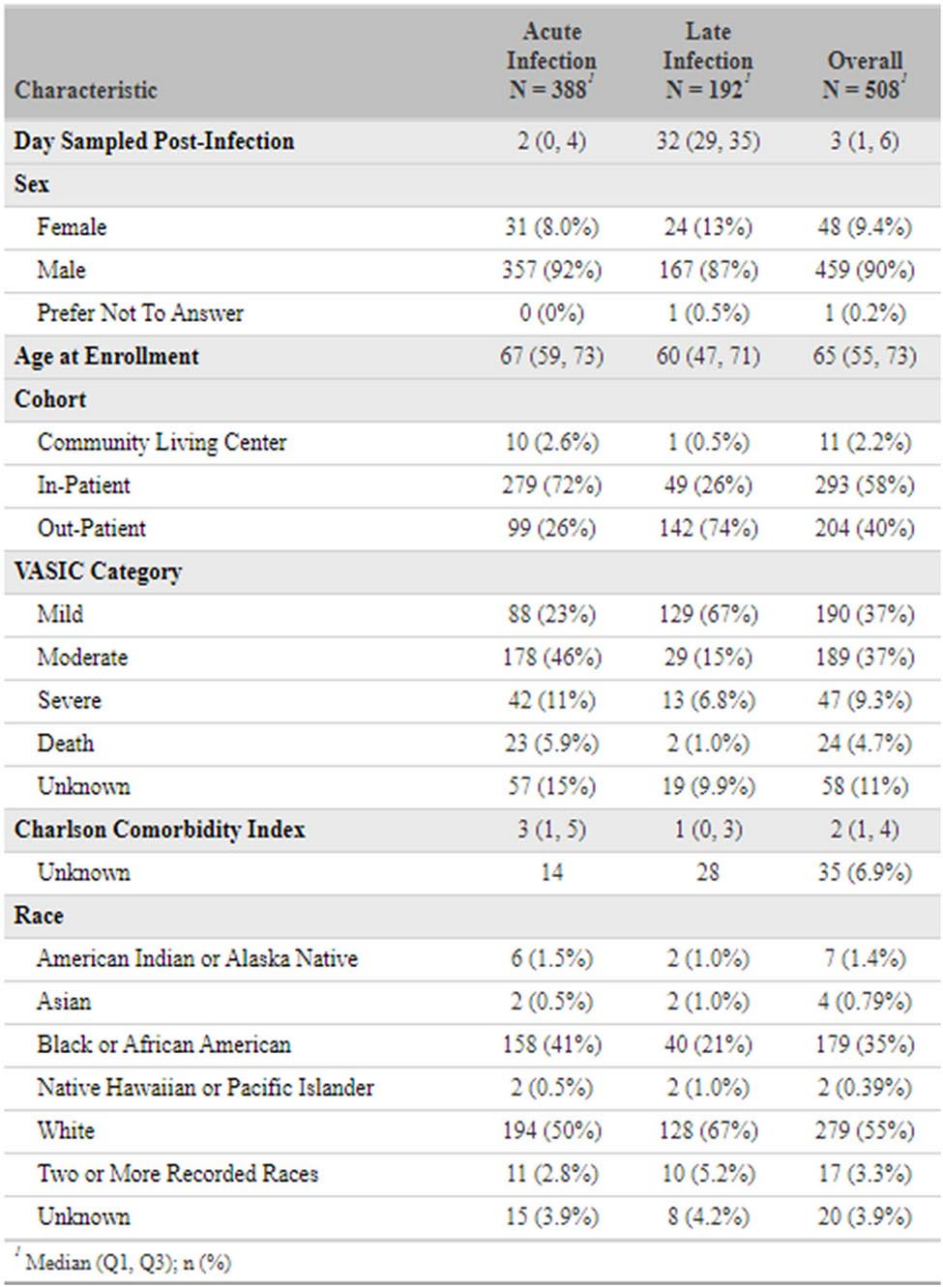
Summary of characteristics of SARS-CoV-2 positive U.S. Veteran participants included in study. Acute infection samples were collected 0-7 days following a positive SARS-CoV-2 test. Late infection samples were collected between 14-42 days following a positive test. Continuous variables are summarized by their median and interquartile range. Categorical variables are summarized by count and percent of samples. Charlson Comorbidity Index excludes age from the calculation.

**Table 2.**
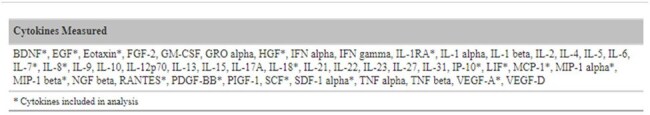
Cytokines assessed in the 45-plex assays. The subset that had less than 20% missingness of concentration values were included in analyses.

**Methods:**

Using a SARS-CoV-2 positive subset (N = 508) of the EPIC^3^ study, a prospective, observational U.S. Veteran cohort (Table 1), we analyzed 18 multiplexed cytokines measured from peripheral blood (Table 2) during acute (0-7 days, N = 388) and late (14-42 days, N = 192) infection stages with COVID-19 severity and Long COVID outcomes. COVID-19 severity was categorized using the Veterans Affairs Severity Index for COVID-19 (VASIC) (‘Mild’, ‘Moderate’, ‘Severe/Death’) as the maximum severity observed within 60 days post-enrollment. Trends between cytokine concentration and severity were assessed using Jonckheere-Terpstra tests. Associations between cytokine concentrations and pairwise VASIC classifications and Long COVID outcomes (PROMIS fatigue 6a, PROMIS cognition 4a, and mMRC Dyspnea scale) at 3 months (median = 101 days, IQR = 90-119.5 days) post-infection were estimated using logistic regression adjusted for age, sex, and Charlson Comorbidity Index.

**Table 3.**
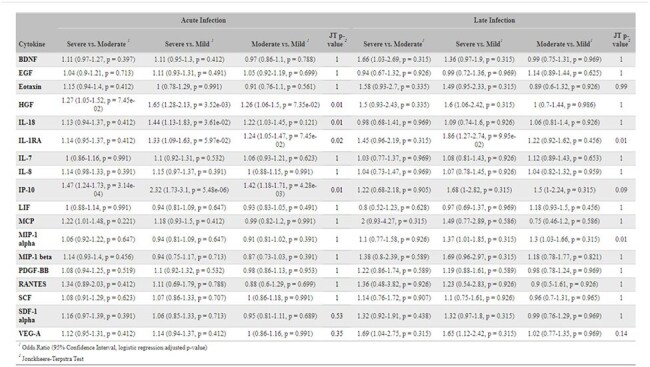
Logistic regression results, with Jonckheere-Terpstra trend tests of cytokine concentration on COVID-19 severity classification.

Models were fit separately on acute stage infection samples and late stage infection samples. Odds of higher disease severity are compared for a 2-fold difference in cytokine concentration in pg/ml. Logistic regression results are adjusted for age, sex, and Charlson Comorbidity Index; cytokine concentrations are adjusted for batch effects and missing values were multiple-imputed. False discovery rate controlled with Benjamini-Hochberg procedure for logistic regression within each symptom at each infection stage; q-values reported. Jonckheere-Terpstra trend test p-values adjusted for multiple comparisons with Bonferroni correction at each infection stage.

**Table 4.**
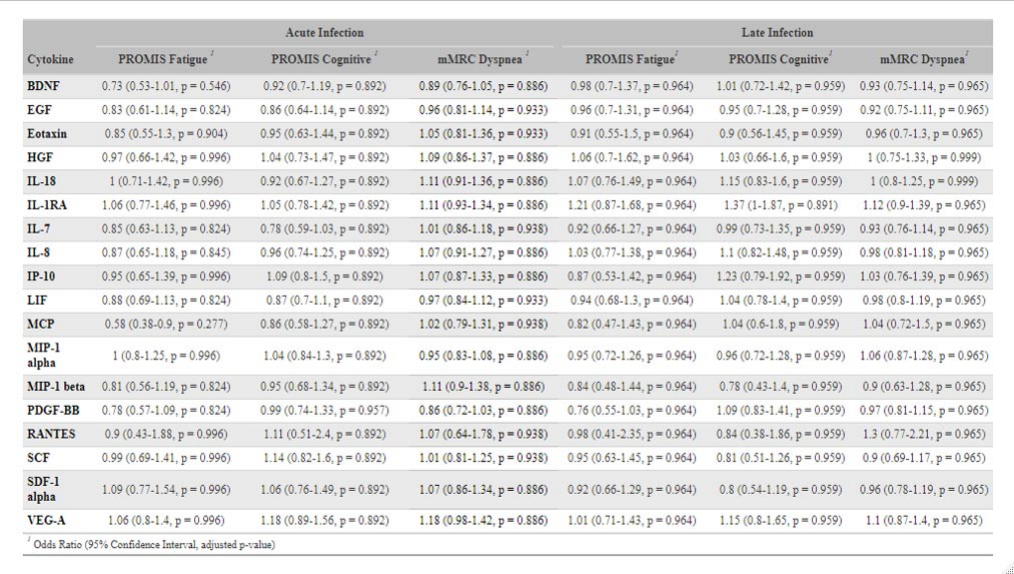
Results of logistic regression of cytokine concentration on Long COVID outcomes.

Models were fit separately on acute infection samples and late infection samples. PROMIS Fatigue was dichotomized as >60 vs < = 60, PROMIS Cognition was dichotomized as < 40 vs. >= 40, and mMRC scale was dichotomized as 1+ vs. 0. Odds of having more severe symptoms are compared for a 2-fold difference in cytokine concentration in pg/ml. Logistic regression results are adjusted for age, sex, and Charlson Comorbidity Index; cytokine concentrations are adjusted for batch effects and missing values were multiple-imputed. False discovery rate controlled with Benjamini-Hochberg procedure within each symptom and at each infection stage; q-values reported.

**Results:**

We found increased IL-1RA, IL-18, IP-10, and HGF at acute infection with greater COVID-19 severity (p< 0.05); this was consistent with a persistent effect at late infection for IL-1RA and IP-10, though only IL-1RA remained statistically significant (Table 3). MIP-1α had a statistically significant positive trend in severity at only late infection. Estimated associations were strongest when comparing mild to severe VASIC groups in logistic regression models (Table 3). No statistically significant associations were detected between cytokine concentration and any Long COVID outcomes at either infection stage after FDR control (Table 4).

**Conclusion:**

: COVID-19 severity was associated with cytokine responses, with IL-1RA consistently associated through late infection. Understanding these markers during SARS-CoV-2 infection may help prioritize care and resources for U.S. Veterans at high risk of severe COVID-19 outcomes.

**Disclosures:**

All Authors: No reported disclosures

